# Severe hyperlipidemia pancreatitis induced by taking tamoxifen after breast cancer surgery—Case report

**DOI:** 10.3389/fonc.2023.1103637

**Published:** 2023-03-10

**Authors:** Chao Zhai, Xiang Li, Dong Xiao, Liang Chen, Chaoyang Wang, MingHua Zheng

**Affiliations:** ^1^ Department of Oncology, Han-Zhong 3201 Hospital Affiliated to Xi'an Jiao-Tong University, Hanzong, China; ^2^ Department of Chemistry, University of Florida, Gainesville, FL, United States

**Keywords:** hyperlipidemia, severe pancreatitis, tamoxifen, breast cancer, endocrine therapy

## Abstract

**Introduction:**

The research investigates the mechanism, diagnosis, treatment, and subsequent endocrine therapy of severe pancreatitis induced by tamoxifen treatment in patients who have undergone breast cancer surgery.

**Case presentation:**

We studied two cases of breast cancer in whom severe acute pancreatitis developed after taking tamoxifen for endocrine therapy in our hospital. A brief literature review was provided to analyze the causes, clinical manifestations, treatment process, and prognosis of severe acute pancreatitis. Both cases involved patients with severe hyperlipidemic pancreatitis. After conservative treatment, none of them died. Pancreatitis did not recur after changing endocrine therapy drugs.

**Discussion/conclusion:**

Endocrine therapy with tamoxifen in breast cancer patients can induce hyperlipidemia, which can subsequently cause severe pancreatitis. The treatment of severe pancreatitis should strengthen the regulation of blood lipids. The application of low-molecular-weight heparin combined with insulin therapy can rapidly lower blood lipids. Involved treatments, including acid suppression, enzyme suppression, and peritoneal dialysis, can accelerate the recovery of pancreatitis and reduce the occurrence of serious complications. Patients with severe pancreatitis should not continue to use tamoxifen for endocrine therapy. To complete follow-up endocrine therapy, switching to a steroidal aromatase inhibitor is better if the situation allows it.

## Introduction

Premenopausal hormone receptor-positive breast cancer patients taking tamoxifen after surgery have become the standard endocrine treatment regimen, with a treatment course of 5–10 years (1, 2). Patients who take tamoxifen for a long time have more common perimenopausal symptoms; however, it can also cause severe abnormal blood lipid metabolism, which in turn induces hyperlipidemic acute pancreatitis (HAP). This disease has a sudden onset, is dangerous and complex, and has a high case-fatality rate, thereby seriously endangering the lives and health of patients. This should arouse great caution in a physician. Here, we retrospectively analyze the relevant case data of our cancer center, review the relevant literature, analyze the possible causes of its occurrence, and summarize the relevant experience of its diagnosis, treatment, and follow-up endocrine therapy.

## Case presentation

Looking back at our hospital records from January 2010 to May 2020, a total of 1,265 patients (1,250 women and 15 men) who took tamoxifen for endocrine therapy after breast cancer surgery were admitted. The total duration of medication ranged from 3 months to 10 years. There was one male case and one female case of hyperlipidemia in acute pancreatitis, with an incidence rate of 0.15%. After receiving active conservative treatment, both patients were cured. Two patients took tamoxifen regularly for 2–3 years until the onset of hyperlipidemic pancreatitis. Those two patients did not receive additional drug therapy. They had no history of hyperlipidemia, pancreatitis, or biliary system disease, no obesity at the time of onset, and a normal body mass index (BMI).

### Case 1

**Figure 1:**
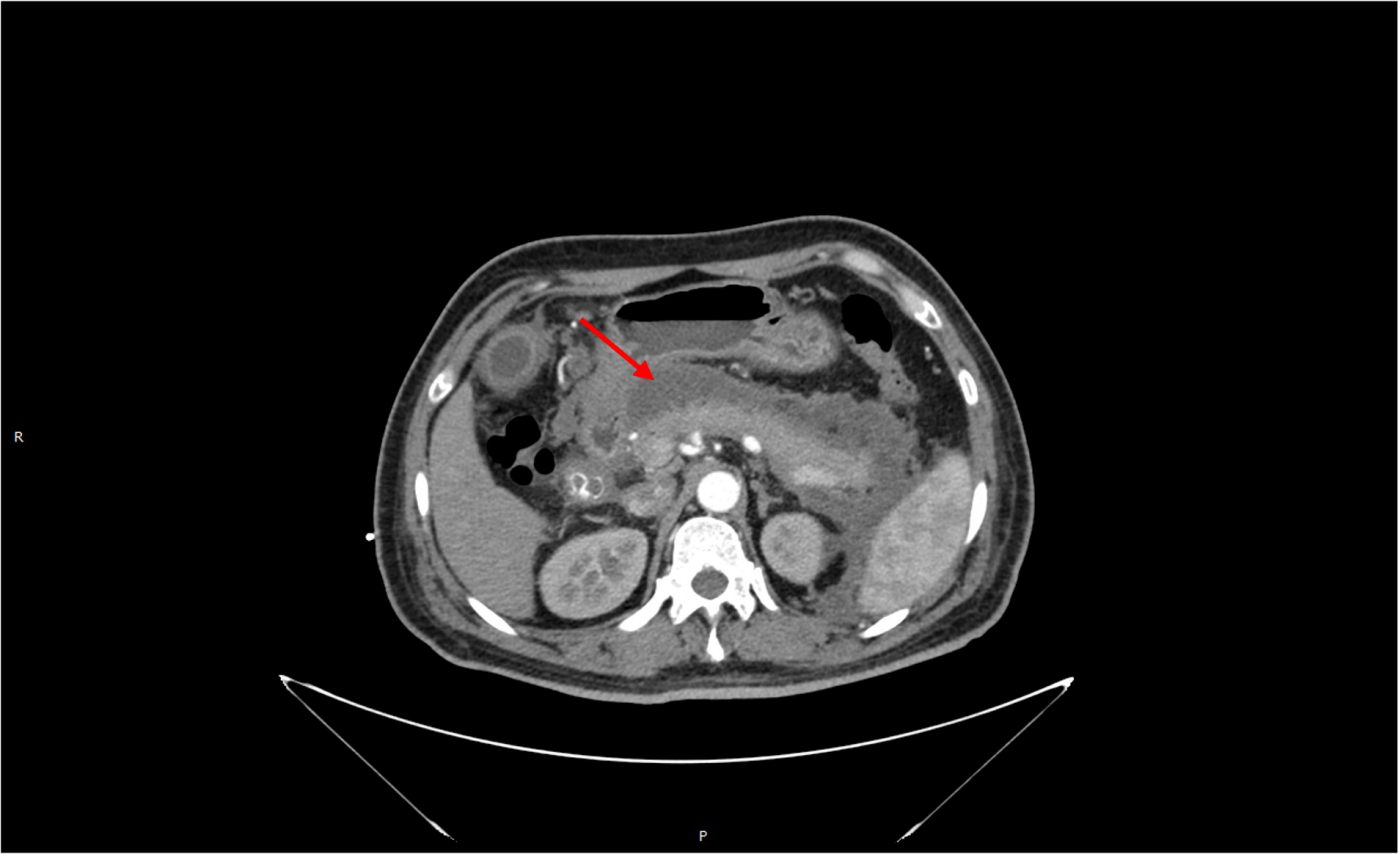
Massive peripancreatic exudate with pancreatic necrosis.

A 46-year-old male patient was admitted to the emergency department on 22 October 2017, due to “persistent left upper abdominal pain for 10 h.” Past history: In 2014, a “modified radical mastectomy” was performed for “right-sided breast cancer.” Medical examination: right invasive breast cancer invaded the nipple with visible nerve, vascular invasion, and intravascular tumor thrombus. No cancer metastasis was found in 39 axillary lymph nodes. Immunohistochemistry: ER (3+, 90%), PR (3+, 85%), Her-2 (−), Ki-67 (40%). After surgery, eight cycles of chemotherapy were used based on the EC-T regimen. After chemotherapy, the patient continued taking tamoxifen (20 mg/day) for endocrine therapy for a total duration of 36 months. The patient has no history of diabetes, hyperlipidemia, pancreatitis, or biliary system disease. The patient has a regular regimen without binge drinking, alcohol consumption, or other triggers before the onset of the disease. Physical examination on admission: T37.3°C, R30 beats/min, P110 beats/min, BP120/80 mmHg, BMI 23.51. Breath sounds in both lungs were thick, with a small number of moist rales heard in both lower lungs. There is no apparent cardiac abnormality on examination. The abdomen was slightly swollen, giving whole-body positive abdominal tenderness on physical examination. The subxiphoid tenderness and tenderness in the left upper abdomen area were significantly noticed, with no rebound tenderness. The patient was negative for Murphy’s sign, with no mobile dullness or weak bowel sounds. Hematological parameters of the patient are listed here: blood amylase 1,541 U/L, lipase 1,574 U/L, triglycerides 91.7 mmol/L, total cholesterol 12.14 mmol/L, blood calcium 1.74 mmol/L, blood sugar 13.6 mmol/L, white blood cells 16.5 × 109/L, neutrophils at 90%, and hematocrit at 50%. Chest and abdomen CT examinations (see **Figure 1**): bilateral lower lung inflammation and bilateral pleural effusion were observed. Acute necrotizing pancreatitis with massive peripancreatic exudation was diagnosed. The abdominopelvic effusion and fatty liver were found, but there were no abnormalities in the biliary system. Liver function test results showed no abnormalities in bilirubin or transaminases. The patient was considered to have acute severe pancreatitis induced by severe hyperlipidemia, excluding biliary pancreatitis and other factors.

Treatment process: 1. conventional treatment such as dietary suppression, gastrointestinal decompression, infection prevention, fluid replacement, volume expansion, and fluid resuscitation; 2. treatment with acid suppression (proton pump inhibitor), enzyme suppression (growth inhibitor or octreotide), and protease inhibitor (ustekin) to inhibit gastric acid and pancreatic juice secretion and suppress inflammatory response in the body; 3. for severe hyperlipidemia, treatment with insulin combined with low molecular heparin: insulin was continuously micropumped to control blood glucose between 5 and 8 mmol/L, and low molecular heparin 6150u was administered subcutaneously twice a day, after which the patient’s triglycerides and total cholesterol decreased to normal on the sixth day after admission. 4. A peritoneal dialysis tube was placed under local anesthesia on the third day after admission for abdominal septal compartment syndrome. A large amount of dark brown, turbid fluid was released, and continuous peritoneal dialysis was performed to reduce intra-abdominal pressure and effectively remove inflammatory factors from the body. 5. The patient’s condition stabilized after one week, and a jejunal nutrition tube was placed intranasally under intervention with Chinese herbal medicine “Qingyi Decoction” (clear pancreas) injected. After intestinal function was restored, enteral nutrition was started, and a probiotic injection was given to prevent severe secondary infections induced by the displacement of intestinal flora.

**Figure 2:**
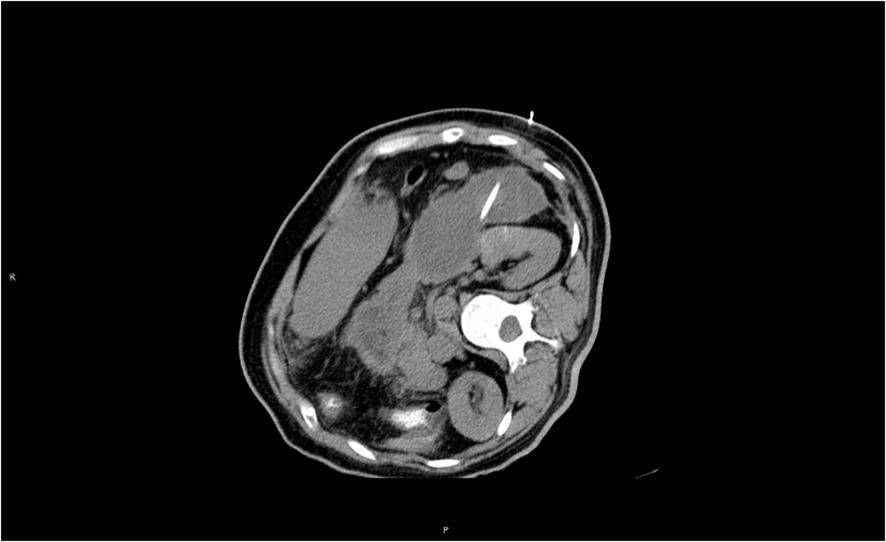
Peripancreatic fluid with infection, CT-guided puncture, and drainage was performed.

The patient continued to experience upper abdominal distension and pain with fever in the fourth week after admission. A repeat CT examination suggested peripancreatic fluid infection with multiple small bubbles and calcitoninogen 13.5 ng/ml. Percutaneous puncture and drainage were performed under CT guidance (see **Figure 2**). Selected antibiotic treatment was taken based on the result of the drug sensitivity test for the patient. The patient recovered well and was discharged after two weeks with the drainage tube removed. After three months, the patient’s CT was rechecked, and no significant abnormality was found, and the peripancreatic exudate was basically absorbed. Triglycerides and total cholesterol were normal. Since the patient had a normal body shape with no obvious obesity, special diet, or apparent cause of hyperlipidemia, we considered that hyperlipidemia might be related to long-term endocrine therapy with tamoxifen after breast cancer surgery. Hence, we stopped prescribing tamoxifen for the patient. The endocrine therapy regimen was changed to goserelin combined with exemestane and was discontinued after a total of two years of treatment. By following up so far, the patient has had no recurrence of breast cancer metastasis or pancreatitis, and lipid monitoring is within the normal range (**Table 1**).

**Table 1:** Triglycerides and cholesterol level for 24 months after HAP treatment.

	Triglycerides (mmol/L)							Cholesterol (mmol/L)						
Times points afters HAP treatment	1W	3M	6M	9M	12M	16M	24M	1W	3M	6M	9M	12M	16M	24M
case 1	2.84	2.16	1.28	1.56	1.28	1.31	1.82	3.74	4.6	4.91	4.99	3.85	4.89	3.7
case 2	2.46	1.66	1.67	1.86	1.19	1.64	1.3	3.32	4.1	4.26	3.25	2.81	2.7	3.26

### Case 2

A 47-year-old female patient was admitted to the emergency department on 9 March 2020, due to “persistent epigastric pain with vomiting and dyspnea for 1 day.” Past history: she underwent “modified radical surgery for right breast cancer” in 2017 for “right breast cancer.” Medical examination: right invasive breast cancer, no nerve or vascular invasion or intravascular cancer thrombus, 21 axillary lymph nodes without cancer metastasis; immunohistochemistry: ER (3+, 80%), PR (3+, 90%), Her-2 (80%), PR (3+, 90%), Her-2 (−), Ki-67 (10%). According to the TC regimen, the patient was treated with four periods of postoperative chemotherapy and continued to take tamoxifen for endocrine therapy at 20 mg/day without interruption until the end of chemotherapy. She had been taking tamoxifen for 30 months until the onset of the latest symptoms. She had no previous history of diabetes, hyperlipidemia, pancreatitis, or biliary system disease. She had a regular lifestyle and had no triggers such as overeating, alcohol consumption, or seafood consumption before the onset of the disease. Physical examination on admission: T38.5°C, R33 times/min, P120 times/min, BP 90/56 mmHg, BMI 22.59.

**Figure 3:**
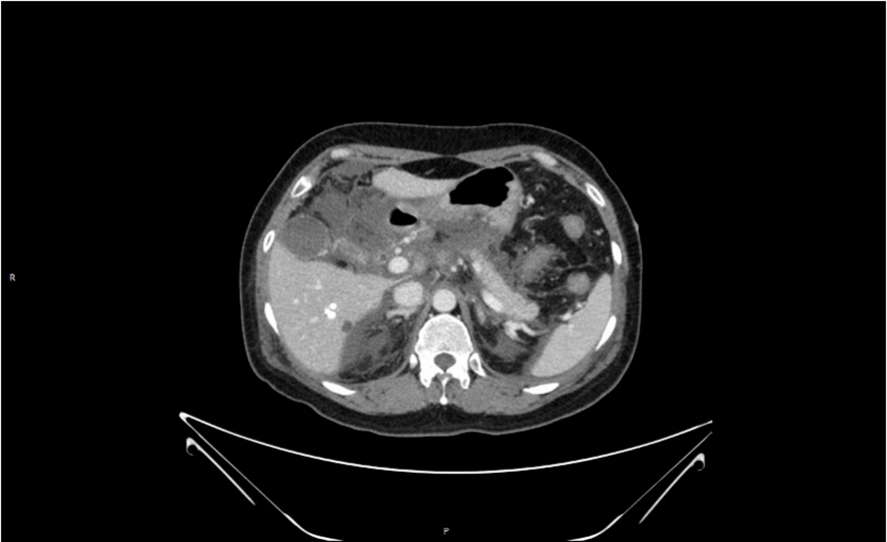
Loss of pancreatic contour, massive oozing from the head of the pancreas, and pancreatic necrosis.

Respiratory sounds were thickened in both lungs, and significant wet rales could be heard in both lower lungs; a cardiac examination did not show any significant abnormalities. The abdomen was slightly distended, and the whole abdomen was positive for pressure pain, especially in the subxiphoid and left epigastrium, with suspicious rebound pain. Murphy’s disease is negative. There is no mobile dullness, and the bowel sounds are weak. Hematological parameters of the patient are listed here: blood amylase 1,248 U/L, lipase 349 U/L, triglycerides 49.8 mmol/L, total cholesterol 8.7 mmol/L, blood calcium 1.80 mmol/L, blood glucose 8.6 mmol/L, white blood cells 18.5 × 109/L, neutrophils 88%, and red blood cell pressure 45%. Liver function indicated no abnormalities in bilirubin and transaminases. CT of the chest and abdomen (see **Figure 3**) shows bilateral lower lung inflammation, bilateral pleural effusion, acute pancreatitis with massive peripancreatic exudate and abdominopelvic effusion, and no abnormalities in the biliary system. The patient was considered to have acute severe pancreatitis, which was induced by severe hyperlipidemia, and biliary pancreatitis and other factors were excluded.

**Figure 4:**
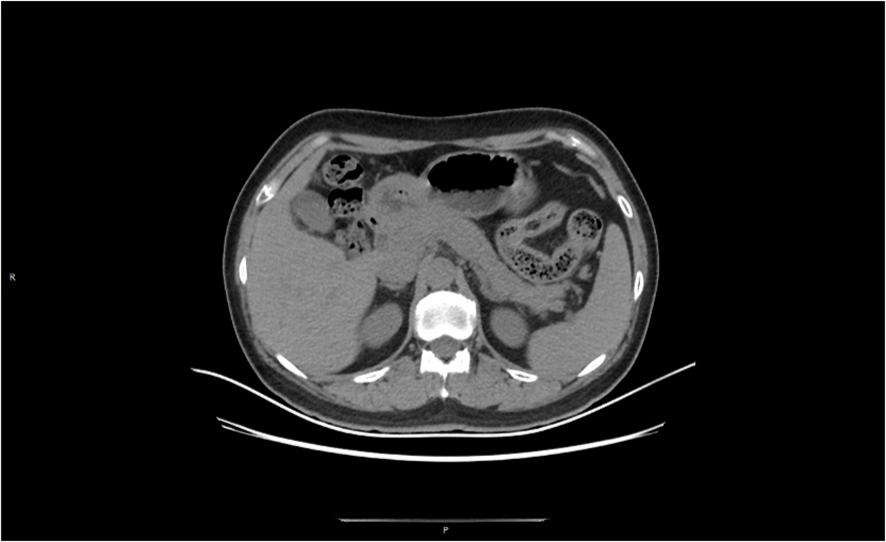
At 11 weeks after treatment, the peripancreatic exudate was largely absorbed and the pancreatic contour was largely restored.

Treatment process: 1. The conventional treatment was the same as in case 1; 2. For severe hyperlipidemia, insulin combined with low-molecular heparin was used. Insulin was continuously micropumped to control blood glucose between 5 and 8 mmol/L, and low-molecular heparin (6,150 u) was administered subcutaneously twice/day. Under treatment, the patient’s triglycerides and total cholesterol fell to normal on the third day after admission. 3. The patient’s abdominal distension became obvious after three days, and a repeat abdominal CT indicated an increase in peritoneal fluid. Therefore, a peritoneal dialysis tube was placed under local anesthesia, releasing a large amount of dark red, turbid fluid. Continuous peritoneal dialysis was performed to reduce intra-abdominal pressure and effectively remove inflammatory factors from the body. 4. The patient’s condition stabilized after one week, and a jejunal nutrition tube was placed nasally under intervention. Chinese herbal medicine “Qingyi Decoction” (clear pancreas) was injected to start enteral nutrition after intestinal function recovered, and a probiotic injection was given to prevent severe secondary infection induced by intestinal flora displacement. 5. The patient was admitted to the hospital in the third week without obvious abdominal distension and abdominal pain, and no positive abdominal signs were seen on physical examination. Routine blood, liver function, and amylase were normal on re-examination. The re-examination CT showed that the peripancreatic fluid was significantly reduced compared with the previous one, and there was no obvious fluid in the abdominopelvic cavity. The patient gradually resumed a transoral diet and was discharged without obvious discomfort. Nearly three months after treatment, the CT was reexamined (see **Figure 4**), and no significant abnormalities were seen. The peripancreatic effusion was basically absorbed. Triglycerides and total cholesterol were normal on reexamination. The condition of the second patient was like that of case 1, with no obvious obesity, a body mass index in the normal range, no special diet, and no obvious trigger for hyperlipidemia. We considered that hyperlipidemia might be related to long-term tamoxifen endocrine therapy after breast cancer surgery, so we stopped using tamoxifen for her. Because she was approaching menopause and the patient and her family members strongly requested an ovariectomy for castration after consultation, a laparoscopic ovariectomy was performed. Exemestane was continued as endocrine therapy after the operation. So far, no recurrence or metastasis of breast cancer has been seen. Pancreatitis, monitor blood lipids are within the normal range (**Table 1**).

We summarized the clinical and lab values for the two cases in **Table 2** and monitored the triglycerides and cholesterol levels for 24 months after HAP treatment and summarized them in **Table 1**.

**Table 2:** The clinical and lab values about the two cases.

	age	FH*	BMI**	Triglycerides (mmol/L)		Cholesterol (mmol/L)	
				Before TAM	After TAM	Before TAM	After TAM
case 1	46	None	23.51	1.68	91.7	4.79	12.14
case 2	47	None	22.59	1.42	49.8	3.71	8.7

*FH, Familial Hypertriglycerid-emia.

**BMI, Body Mass Index.

## Conclusion

### The mechanism of tamoxifen-induced hyperlipidemia

Tamoxifen (TAM), as a selective estrogen receptor modulator, occupies an important position in the endocrine treatment of hormone receptor-positive (HR-positive) breast cancer patients. It is a cornerstone drug in the clinic for endocrine therapy in HR-positive breast cancer patients because of its low price and established efficacy. However, long-term use of tamoxifen can lead to menopausal symptoms and endometrial cancer, which are taken seriously. In contrast, tamoxifen-induced hyperlipidemia is overlooked. Saphner et al. (3) showed that the incidence of fatty liver in patients on long-term tamoxifen was 4.5%. In a study by Akhondi-Meybodi et al. (4), mean triglyceride levels were significantly higher in tamoxifen-induced patients with fatty livers than in normal controls.

Reviewing the relevant literatures (5–7), tamoxifen-caused hyperlipidemia was mainly manifested by a significant increase in serum triglyceride levels, whereas serum cholesterol and ultra-low density lipoprotein levels were unaffected or reduced. In our center, we observed two cases in which triglyceride levels exceeded the normal level by 29 and 53 times, respectively, while cholesterol levels exceeded the normal level by only about two times, which is consistent with literatures. Although there are reports suggesting that hypertriglyceridemia after tamoxifen may be related to preexisting conditions such as diabetes, chronic kidney disease, and nonalcoholic fatty liver disease (8), none of these preexisting diseases were found in our cases. The possible mechanisms behind tamoxifen-induced hyperlipidemia include: 1) Tamoxifen is a selective estrogen receptor modulator, which exerts partial estrogenic effects on lipid metabolism, inhibits post-heparin lysyl lipase (PHLA) activity, inhibits triglyceride lipase, and increases serum triglyceride concentration; 2) Tamoxifen can significantly downregulate the expression and activity of fatty acid synthase (FAS), thereby inhibiting fatty acid β-oxidation; and 3) Tamoxifen can affect the expression of nuclear receptors involved in lipid metabolism (androgen receptor, hepatocyte nuclear factor 4α, sterol regulatory element binding protein-1c), promote fatty acid synthesis, and increase TG levels significantly.

### Treatment of hyperlipidemia pancreatitis

Hyperlipidemic acute pancreatitis (HAP), also known as hypertriglyceridemic pancreatitis, is closely related to serum triglycerides (TG) but not to serum cholesterol (TC) (9). Based on excluding biliary obstruction and other factors inducing acute pancreatitis (AP), the diagnosis of HAP can be confirmed when the fasting blood TG value after admission is over 11.3 mmol/L. HAP can also be diagnosed when the blood TG value is 5.65–11.3 mmol/L with celiac serum presented. Tamoxifen-induced hyperlipidemic pancreatitis has both the general characteristics of acute pancreatitis and its specificity. Therefore, based on the standardized treatment of AP, the key to the treatment of HAP is to rapidly remove the factors causing HL and rapidly reduce the blood TG value.

To summarize the data of this group, we believe that (1) the key to the treatment of this disease is timely and accurate diagnosis and an active search for possible predisposing factors. Therefore, for suspected cases of HAP, in addition to routine CT\MRCP examinations to exclude biliary factors, lipid examinations should be performed, and drug intake history should be asked. If such factors are present, the relevant drugs should be discontinued immediately (2). Rapid and effective reduction of serum TG levels plays a decisive role in the treatment of this disease. The relevant literature reports (10, 11) that the main lipid-lowering measures for HAP are oral lipid-lowering drugs (fibrates), blood purification, plasma exchange combined with lipid adsorption, and intravenous heparin combined with insulin. Analyzing the treatment of this group of cases, we used a comprehensive treatment plan of diet abstinence, strict fat-free total parenteral nutrition, and insulin combined with subcutaneous injection of low-molecular heparin, which could rapidly and effectively lower the serum TG level to normal within 3–6 days of admission. Oral lipid-lowering drugs, hemodialysis, and plasma exchange were not used in either case. This method is simple, inexpensive, safe, effective, and easy to promote clinically. However, for patients with early combined multi-organ failure, we believe that hemofiltration combined with lipid adsorption is also a practical and effective treatment (3). For the treatment of peritoneal septal compartment syndrome in the early stages of severe pancreatitis, our center routinely places peritoneal dialysis tubing (12) and continuous peritoneal dialysis to reduce intra-abdominal pressure and effectively remove inflammatory factors from the body to reduce the occurrence and development of MODS (4). For the treatment of peripancreatic infection in the middle and late stages of the disease, we mostly use CT-guided percutaneous puncture placement of negative pressure flushing and drainage (13), combined with sensitive antibiotic treatment. Such a strategy can effectively control infection, avoid traditional open abdominal debridement and drainage, and reduce the incidence of postoperative gastrointestinal injury and fistulas (5). For the comprehensive treatment of severe pancreatitis, we believe that, on the basis of traditional treatment, a jejunal nutrition tube should be placed as early as possible, with external application of mannitol and injection of clear pancreatic soup through the nutrition tube. After the recovery of intestinal function, enteral nutrition should be started as early as possible to protect the intestinal mucosa to prevent severe secondary infections induced by intestinal flora displacement.

### Endocrine therapy for breast cancer

Endocrine therapy has become an integral and important part of the comprehensive treatment of hormone receptor-positive breast cancer patients. Commonly used drugs include selective estrogen receptor modulators (tamoxifen, toremifene, and fulvestrant), nonsteroidal aromatase inhibitors (letrozole and anastrozole), steroidal aromatase inhibitors (exemestane), etc. Among them, tamoxifen can exert a unique lipoprotective advantage due to its weak estrogenomimetic effect (14), and it is used as the drug of choice for endocrine therapy in premenopausal HR-positive breast cancer patients in clinical practice because of its established efficacy and affordability. However, its lipoprotective effect is limited to lowering serum cholesterol (TC) levels and ultra-low-density lipoprotein (LDL-C) levels but can significantly increase serum triglyceride (TG) levels (15). Some authors suggest that after tamoxifen-induced hyperlipidemic pancreatitis, letrozole can be replaced to complete subsequent endocrine therapy (16, 17). However, numerous publications suggest that nonsteroidal aromatase inhibitors such as letrozole can induce severe dyslipidemia (hypercholesterolemia) and should be closely monitored during clinical use (14, 15, 18).

Therefore, by summarizing our data and reviewing the relevant literature and guidelines (14, 15, 18, 19), we have the following conclusions: (1) When breast cancer patients undergo endocrine therapy, lipid levels should be routinely monitored, with preoperative levels as the baseline standard, and tested every 6–12 months. If combined with high-risk factors or dyslipidemia, lipid-modifying drugs should be given promptly for intervention. (2) Complications such as tamoxifen-induced hypertriglyceridemia and fatty liver are often overlooked, which often occur after 12 months of tamoxifen treatment, and some patients have life-threatening severe pancreatitis induced by severe hypertriglyceridemia. Only sporadic cases have been reported both domestically and internationally, and the causes of pathogenesis, treatment options, and follow-up endocrine therapy options have not been explored in depth (16, 17). The two patients in our group did not routinely monitor their lipids after surgery, and both had severe hyperlipidemic pancreatitis complicated by tamoxifen treatment for more than two years. Although they recovered after active treatment, it should be given our great attention that when using tamoxifen, we should not assume that it has lipoprotective effects because it can lower TC and LDL-C levels and neglect monitoring of lipids. (3) Toremifene lowers TC and LDL-C levels comparable to tamoxifen and does not affect TG levels. Exemestane has comparable effects on TC and LDL-C levels to tamoxifen, can effectively lower TG levels, and can be used safely in postmenopausal patients. Therefore, toremifene and steroidal aromatase inhibitors (exemestane) are alternative drugs that can be used in patients with severe hypertriglyceridemia or hyperlipidemic pancreatitis to complete subsequent endocrine therapy. However, non-steroidal aromatase inhibitors (letrozole and anastrozole) should not be use. (4) When endocrine therapy is administered to a special group of male breast cancer patients, more attention should be paid to the occurrence of such complications. In cases of uncontrollable hyperlipidemia, it is recommended to change to toremifene or use goserelin combined with exemestane treatment. Patients should be encouraged to quit smoking and alcohol, exercise, change bad habits, and monitor lipid levels closely.

## Data availability statement

The original contributions presented in the study are included in the article/supplementary material. Further inquiries can be directed to the corresponding author.

## Ethics statement

The studies involving human participants were reviewed and approved by the Ethical Committee of Han-Zhong 3201 Hospital Affiliated to Xi’an Jiao-Tong University. The patients/participants provided their written informed consent to participate in this study. Written informed consent was obtained from the individual(s) for the publication of any potentially identifiable images or data included in this article.

## Author contributions

MZ: the conception or design of the work. CZ, XL, DX, LC, and CW: the acquisition, analysis, or interpretation of data for the work. CZ and MZ: drafting the work or revising it critically for important intellectual content. CZ and MZ: final approval of the version to be published. All authors contributed to the article and approved the submitted version.

## References

[1] Burstein HJ Temin S Anderson H Buchholz TA Davidson NE Gelmon KE . Adjuvant endocrine therapy for women with hormone receptor-positive breast cancer: american society of clinical oncology clinical practice guideline focused update. J Clin Oncol (2014) 32(21):2255–69. doi: 10.1200/JCO.2013.54.2258 PMC4876310 24868023

[2] Davies C Pan H Godwin J Gray R Arriagada R Raina V . Long-term effects of continuing adjuvant tamoxifen to 10 years versus stopping at 5 years after diagnosis of oestrogen receptor-positive breast cancer: ATLAS, a randomised trial. Lancet (2013) 381(9869):805–16. doi: 10.1016/S0140-6736(12)61963-1 PMC3596060 23219286

[3] Saphner T Triest-Robertson S Li H Holzman P . The association of nonalcoholic steatohepatitis and tamoxifen in patients with breast cancer. Cancer (2009) 115(14):3189–95. doi: 10.1002/cncr.24374 19484789

[4] Akhondi-Meybodi M Mortazavy-Zadah MR Hashemian Z Moaiedi M . Incidence and risk factors for non-alcoholic steatohepatitis in females treated with tamoxifen for breast cancer. Arab J Gastroenterol (2011) 12(1):34–6. doi: 10.1016/j.ajg.2011.01.003 21429453

[5] Lelliott CJ Lopez M Curtis RK Parker N Laudes M Yeo G . Transcript and metabolite analysis of the effects of tamoxifen in rat liver reveals inhibition of fatty acid synthesis in the presence of hepatic steatosis. FASEB J (2005) 19(9):1108–19. doi: 10.1096/fj.04-3196com 15985534

[6] Zhao F Xie P Jiang J Zhang L An W Zhan Y . The effect and mechanism of tamoxifen-induced hepatocyte steatosis *in vitro* . Int J Mol Sci (2014) 15(3):4019–30. doi: 10.3390/ijms15034019 PMC3975381 24603540

[7] Lee MH Kim JW Kim JH Kang KS Kong G Lee MO . Gene expression profiling of murine hepatic steatosis induced by tamoxifen. Toxicol Lett (2010) 199(3):416–24. doi: 10.1016/j.toxlet.2010.10.008 20937368

[8] Isobe H Shimoda M Kan Y Tatsumi F Katakura Y Kimura T . A case of tamoxifen-induced hypertriglyceridemia monitoring the changes in lipoprotein fractions over time. BMC Endocr Disord (2021) 21(1):115. doi: 10.1186/s12902-021-00780-z 34107939 PMC8191117

[9] Yeh JH Chen JH Chiu HC . Plasmapheresis for hyperlipidemic pancreatitis. J Clin Apher (2003) 18(4):181–5. doi: 10.1002/jca.10063 14699594

[10] Wang G Sun B Jiang H . Advances in research on acute pancreatitis induced by hyperlipidemia. Chin J Gen Surg (2005) 14(11):857–9. doi: 10.3969/j.issn.1005-6947.2005.11.016

[11] Shi X Wang G Liu Y Wang Y Liu K . Treatment of hyperlipidemic pancreatitis. Chin J Hepatobiliary Surg (2011) 17(11):949–52. doi: 10.3760/cma.j.issn.1007-8118.2011.11.026

[12] Ming L Liu X Xiang J Zhai C . Clinical study of early peritoneal dialysis in severe acute pancreatitis. Chin J Curt Adv Gen Surg (2012) 15(2):164–6. doi: 10.3969/j.issn.1009-9905.2012.02.029

[13] Zhai C Liu X Fu W Zheng M Xiao D Liang M . Treatment of severe acute pancreatitis using CT guided percutaneous puncture and negative pressure catheter drainage combined with peritoneal dialysis. Chin J Hepatobiliary Surg (2015) 21(5):317–20. doi: 10.3760/cma.j.issn.1007-8118.2015.05.009

[14] Breast International Group 1-98 Collaborative G Thurlimann B Keshaviah A Coates AS Mouridsen H Mauriac L . A comparison of letrozole and tamoxifen in postmenopausal women with early breast cancer. N Engl J Med (2005) 353(26):2747–57. doi: 10.1056/NEJMoa052258 16382061

[15] Hozumi Y Suemasu K Takei H Aihara T Takehara M Saito T . The effect of exemestane, anastrozole, and tamoxifen on lipid profiles in Japanese postmenopausal early breast cancer patients: final results of national surgical adjuvant study BC 04, the TEAM Japan sub-study. Ann Oncol (2011) 22(8):1777–82. doi: 10.1093/annonc/mdq707 21285133

[16] Li H Sheng C Hao Y . A case of acute pancreatitis caused by hyperlipidemia induced by tamoxifen. Chin J Pancreatol (2018) 18(6):398. doi: 10.3760/cma.j.issn.1674-1935.2018.06.010

[17] Tey TT Maung AC Lim KW Hsiang JC . Acute pancreatitis caused by tamoxifen-induced severe hypertriglyceridemia after 4 years of tamoxifen use. ACG Case Rep J (2019) 6(2):1–3. doi: 10.14309/crj.0000000000000025 PMC6657993 31157284

[18] Ma F Xu BH Shao ZM Experts Committee of Chinese Guideline on the Breast Cancer F-u, Concomitant Diseases Comprehensive M . [Comprehensive management guideline for breast cancer follow-up and concomitant diseases]. Chin J Oncol (2019) 41(1):29–41. doi: 10.3760/cma.j.issn.0253-3766.2019.01.006 30678414

[19] Tian L Xu B . Comparative study of the effects of toremifene and tamoxifen on blood lipid levels in breast cancer. Pract J Cancer (2004) 19(5):520–2. doi: 10.3969/j.issn.1001-5930.2004.05.024

